# Operation for Tuberculous Glands in the Neck

**Published:** 1901-10-12

**Authors:** 


### OPERATION FOR TUBERCULOUS GLANDS IN THE NECK.

Dealing with the surgical treatment of tuberculous glands in the neck, Mr. Butlin[1] gives a warning which is certainly not altogether out of place at the present day, in regard to the sometimes serious and embarrassing nature of the operation for excision of such glands. To get out a large number of glands through a small incision in an inconvenient situation is difficult enough ; but how much more difficult when the glands are widely scattered, softening, breaking down, adherent to and sometimes incorporated with the surrounding structures, which may be the main nerves or vessels of the neck ! "I have heard," he says, "of more than one fatal accident in course of these operations, and I am surprised that accidents are not more frequent. The worst thing which has yet happened to me has been to have to ligature the common carotid artery, and that was serious enough." If a sudden and severe hæmorrhage happens at the bottom of a deep hole, where one is operating through a wound made as small as possible so as not to impair the appearance of the patient in after life, the difficulty in dealing with it is very great. In such a case one should instantly plug the bottom of the wound with gauze, and then make a much larger opening. It is no longer a question of appearance, but of life and death. Then there is another danger in these operations which must always be borne in mind, and that is the fear of damage to the spinal accessory nerve. "To tell the truth," says Mr. Butlin, "there are many capital operations in surgery which I would rather face than an operation for the removal of tuberculous cervical glands, and I am always pleased when it is over."

[1] Clin. Journ., Sept. 11.

